# Bradycardia, Renal Failure, Atrioventricular Nodal Blockade, Shock, and Hyperkalemia (BRASH) Syndrome: A Rising Entity of Severe Bradycardia

**DOI:** 10.7759/cureus.35620

**Published:** 2023-02-28

**Authors:** Choong Tatt Ng, Kai Xiong Lim, Khang Ning Loo

**Affiliations:** 1 General Medicine, Sengkang General Hospital, Singapore, SGP

**Keywords:** smart devices, acute kidney injury, bradycardia, hyperkalaemia, brash syndrome

## Abstract

Bradycardia, renal failure, atrioventricular nodal blockade, shock, and hyperkalemia (BRASH) syndrome is an entity recently coined to describe this clinical pentad. Although the condition is rare, early recognition is paramount. It ensures prompt appropriate intervention is administered, as conventional management for bradycardia as guided by advanced cardiac life support (ACLS) is ineffective in the BRASH syndrome. Here, we describe a case of an elderly lady with hypertension and chronic kidney disease presenting to the emergency department with dyspnoea and confusion. She was found to have bradycardia, hyperkalemia, and acute kidney injury. Notably, she had recent changes in her medications in view of poorly controlled hypertension two days before the presentation. Her Bisoprolol 5mg every morning was changed to Carvedilol 12.5mg twice daily, and Amlodipine 10mg every morning was changed to Nifedipine long-acting 60mg twice daily. Initial treatment with atropine for bradycardia was ineffective. However, when the BRASH syndrome was identified and treated, the patient’s condition improved, and she averted complications such as multiorgan failure without the need for dialysis or cardiac pacing. Early detection of bradycardia via smart devices could be considered in patients at higher risk of BRASH syndrome.

## Introduction

Bradycardia, renal failure, atrioventricular nodal blockade, shock, and hyperkalemia (BRASH) syndrome is a relatively new entity first described by Farkas in 2016 [1]. It remains an under-recognized and poorly diagnosed condition [2]. We discuss a case of an elderly patient with predisposing factors to BRASH syndrome presenting to the emergency department with bradycardia. We review the importance of prompt recognition of the BRASH syndrome and how it affected the management and outcome of the patient.

## Case presentation

A 71-year-old Chinese lady presents acutely to the emergency department for confusion and dyspnoea. Her significant past medical history includes diabetes mellitus, hypertension, hyperlipidemia, stage G1A3 chronic kidney disease secondary to diabetic kidney disease, and non-alcoholic steatohepatitis cirrhosis.

She had recently visited her primary care physician two days before the emergency department attendance for a routine review of her chronic medical conditions. Notably, her antihypertensive medications were adjusted in view of poorly controlled hypertension. Her Bisoprolol 5mg every morning was changed to Carvedilol 12.5mg twice daily, and Amlodipine 10mg every morning was changed to Nifedipine long-acting 60mg twice daily. Her other chronic medications were otherwise unchanged. They included Aspirin 100mg every morning, Atorvastatin 40mg every night, Gliclazide modified release 60mg every morning, Famotidine 20mg, Linagliptin 5mg every morning, Metformin 850mg three times daily, Spironolactone 12.5mg every morning and Valsartan 160mg twice daily.

On arrival at the emergency department, the patient was bradycardic but normotensive. Her heart rate was 38 beats per minute (BPM), her blood pressure was 142/117 mmHg, and her oxygen saturation was 100% on a 50% venturi mask. She was afebrile. The patient was clinically in fluid overload with signs of bilateral basal crepitations on auscultation of the lungs and pitting edema of the bilateral lower limbs extending to the knees. 

**Figure 1:**
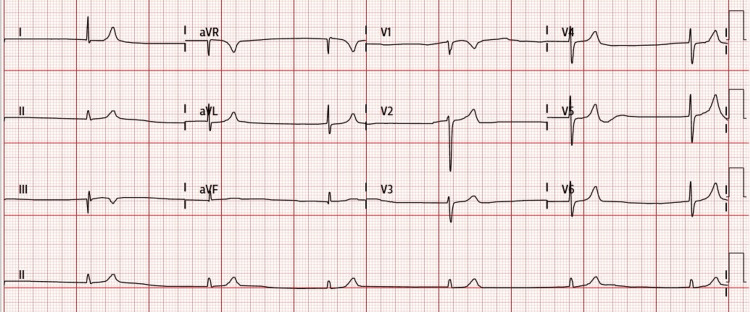
Electrocardiography on presentation showing junctional bradycardia

She was treated for bradycardia initially as per the advanced cardiac life support (ACLS) algorithm. Her initial electrocardiography (ECG) revealed junctional bradycardia with a rate of about 35 BPM (Figure 1). There were no hyperkalemia ECG changes, such as widened QRS complexes and tall tented T waves. Two doses of intravenous atropine 600mcg were given. However, there was no improvement in the bradycardia.

Laboratory investigations subsequently returned and are summarized in Table 1. Notably, there was severe hyperkalemia of 8.2mmol/L, metabolic acidosis, and acute kidney injury. The patient’s creatinine level rose from a baseline of 50µmol/L to 161µmol/L on admission. Serum lactate level was elevated at 9.0 mmol/L from hypoperfusion.

**Table 1:** Laboratory findings on admission

Laboratory findings	Value on admission	Normal Range
Biochemistry		
Urea (mmol/L)	9.1	2.7 – 6.9
Sodium (mmol/L)	127	136 – 146
Potassium (mmol/L)	8.2	3.5 – 5.1
Chloride (mmol/L)	96	98 – 107
Bicarbonate (mmol/L)	14.1	19.0 – 29.0
Creatinine (µmol/L)	161	45 – 84
Lactate (mmol/L)	9.0	0.5 – 2.2
Ketones (mmol/L)	0.2	0.0 – 0.6
Corrected Calcium (mmol/L)	2.39	2.09 – 2.46
Phosphate (mmol/L)	1.24	0.80 – 1.50
Magnesium (mmol/L)	0.59	0.74 – 0.97
Troponin T (ng/L)	15	<20
Full Blood Count		
Haemoglobin (g/dL)	11.8	12.0 – 16.0
White Blood Cell Count (x10^9^/L)	9.89	4.00 – 10.00
Platelet Count (x10^9^/L)	114	140 – 440
Arterial Blood Gas on 50% Venturi Mask		
pH	7.264	7.350 – 7.450
Bicarbonate (mmol/L)	20.0	22.0 – 26.0
PCO2 (mmHg)	44.1	35.0 – 45.0
PO2 (mmHg)	109.0	80.0 – 105.0
O2 Saturation (%)	97.0	95.0 – 98.0

There was prompt recognition of the BRASH syndrome, and that guided the subsequent management of the patient. Five doses of 10ml calcium gluconate 10% (2.3mmol/10ml) solution were given intravenously in total to stabilize the cardiac membranes. The hyperkalemia was treated with intravenous (IV) 10U insulin with 50ml of dextrose 50%, oral sodium zirconium cyclosilicate oral powder 10g, rectal sodium polystyrene sulfonate enema 30g, and salbutamol 0.5% nebulization. IV isotonic sodium bicarbonate 500ml was also given to shift potassium intracellularly and treat the metabolic acidosis. IV furosemide 80mg was given in view of the fluid-overloaded state. An indwelling urinary catheter was inserted, which drained minimal urine. There was a view for dialysis if hyperkalemia persisted despite the measures taken above.

**Figure 2:**
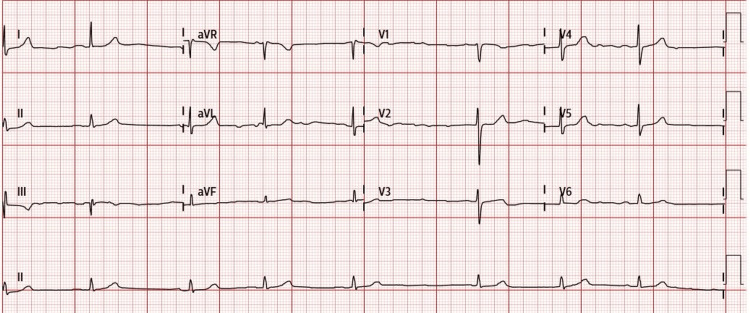
Electrocardiography shows sinus bradycardia and improvement of heart rate to 45 BPM

**Figure 3:**
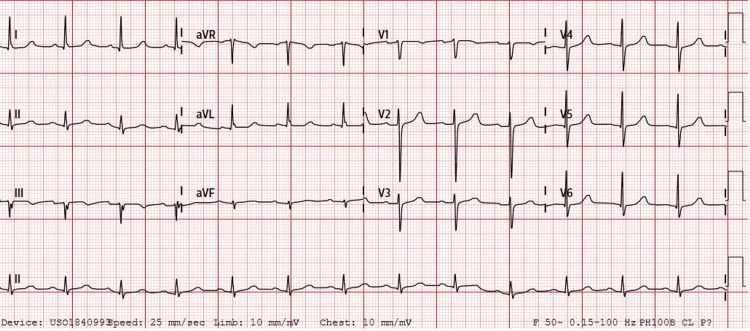
Electrocardiography shows sinus rhythm with a heart rate of about 70 BPM

The patient’s bradycardia resolved, and her hemodynamics improved after aggressive treatment of the hyperkalemia. Serum potassium level normalized within 17 hours from the presentation to the hospital. Figure 2 is an ECG performed demonstrating improvement of the bradycardia to a rate of about 45 BPM as hyperkalemia was being treated. Figure 3 is an ECG performed when hyperkalemia resolved, demonstrating normal sinus rhythm at a heart rate of about 70 BPM. The patient did not require the administration of a positive chronotropic agent such as epinephrine. Her renal function recovered with good production of urine and improvement of creatinine levels without the need for dialysis.

On discharge, the patient’s Carvedilol dose was decreased to 6.25mg twice daily, and Nifedipine long-acting was decreased to 30mg twice daily. Her serum creatinine improved to 89µmol/L two months post-discharge.

## Discussion

The BRASH syndrome is the outcome of a synergistic effect of hyperkalemia and atrioventricular (AV) nodal blocking agents in causing bradycardia [3]. Bradycardia causes a decrease in cardiac output, which, in turn, results in kidney injury via decreased renal perfusion. Impaired renal function exacerbates the hyperkalemia resulting in further worsening of the bradycardia [1]. This results in a vicious cycle of chain reactions that could be life-threatening. 

ECG changes can range from sinus bradycardia to junctional bradycardia and all degrees of AV blocks [4]. ECG findings in BRASH syndrome typically do not show hyperkalaemia-associated changes such as tall tented T waves and widened QRS complexes [1,5], similar to our patient’s case. 

Culprit medications involved in AV node blockage often involve beta blockers and non-dihydropyridine calcium channel blockers such as verapamil and diltiazem. Dihydropyridine calcium channel blockers can occasionally cause bradycardia and have the potential to precipitate BRASH syndrome [1,3,6]. A recent case report on BRASH syndrome reports possible precipitants of an increase in dosage of Nifedipine and a change of a beta-1 selective beta blocker to a non-selective one [4]. It was postulated that nifedipine could have played a role in AV node suppression [7]. This is similar to our patient’s case, where beta-1 selective bisoprolol was changed to non-selective carvedilol, and Amlodipine 10mg once daily was changed to nifedipine long-acting 60mg twice daily.

The BRASH syndrome is more common in the elderly with a history of cardiac and renal impairment [3]. Triggers of BRASH syndrome include dehydration and an increase in the dosage of medications that can cause hyperkalemia and kidney injury, for example, potassium-sparing diuretics [3]. Medications that could increase the risk of BRASH syndrome include angiotensin-converting enzyme inhibitors or angiotensin-receptor blockers through hyperkalemia and kidney injury [1]. Close attention should be paid to high-risk patients when initiating or increasing dosages of AV nodal blocking agents, as in our patient. 

Treating BRASH syndrome involves immediately addressing hyperkalemia. Prompt administration of IV calcium to stabilize the cardiac membrane, nebulized Salbutamol and IV insulin with dextrose to shift potassium into cells, and potassium-wasting diuretics for potassium excretion [3]. Isotonic bicarbonate can be considered to treat metabolic acidosis; the resolution of acidosis has the added effect of the intracellular shift of potassium [3]. In cases of hypovolemia, fluid resuscitation should be given. However, care must be taken as patients with BRASH syndrome are at high risk of fluid overload since acute kidney injury and low urine output are known complications. Dialysis can be considered in severe kidney injury and refractory hyperkalemia. 

IV epinephrine can be considered for bradycardia to increase heart rate and cardiac output. It can also cause intracellularly potassium shift [3]. The inability to recognize the BRASH syndrome and strict adherence to ACLS protocol for bradycardia will result in a delay in treatment. 

The bradycardia from AV nodal blockade is the likely initial trigger for the cascade of metabolic complications. Thus, closer attention should be given to monitoring for bradycardia in patients at higher risk of BRASH syndrome. Smart devices equipped with cardiac monitoring have seen increased accuracy and affordability. Smartwatches have previously been reported to detect ventricular tachycardia [8,9]. The accuracy of various wrist-worn wearable devices for the detection of paroxysmal supraventricular tachycardia was reported at 87%-100% [10]. Early detection of bradycardia via smart devices could allow for prompt diagnosis and treatment of BRASH syndrome before patients develop further complications.

## Conclusions

BRASH syndrome remains a poorly recognized entity that has significant implications for a patient’s management. Further studies to identify the variable presentations and to create reliable diagnostic criteria could help to increase physicians’ understanding and appreciation of this potentially life-threatening condition. Careful attention should also be given to patients at higher risk of BRASH syndrome when using medications associated with the condition. Our case demonstrated how early recognition and treatment of BRASH syndrome could prevent serious complications, including multi-organ failure and death. Smart devices could have a role in the early detection of BRASH syndrome in high-risk populations.
